# Effect of training based on health belief model and behavioral intention on improving dental and oral self-care behavior in 9–12-year-old Iranian female students

**DOI:** 10.1186/s12903-022-02552-0

**Published:** 2022-11-19

**Authors:** Fatemeh Mohammadkhah, Ali Ramezankhani, Abolfazl Atashpoosh, Farzaneh Ahmady Moghadam, Maryam Bakhtiar, Ali Khani Jeihooni

**Affiliations:** 1 Department of Community Health, Child Nursing and Aging, Ramsar School of Nursing, Babol University of Medical Sciences, Babol, Iran; 2 Department of Public Health, School of Public Health and Safety, Shahid Beheshti University of Medical Sciences, Tehran, Iran; 3 Department of Psychology, Lahijan Branch, Islamic Azad University, Lahijan, Iran; 4 Department of Psychology, Islamic Azad University, Rasht Branch, Rasht, Iran; 5 Department of Dental Public Health, School of Dental, Shiraz University of Medical Sciences, Shiraz, Iran; 6 Department of Public Health, School of Health, Nutrition Research Center, Shiraz University of Medical Sciences, Shiraz, Iran

**Keywords:** Health Education, Dental, Health Belief Model, Behavior, Women, Self-care, Health-Related Behaviors, Students

## Abstract

**Background:**

Training dental and oral health behaviors by using appropriate training models and theories is an important issue in preventing dental and oral diseases. the present study aimed to investigate the effect of training based on the health belief model and behavioral intention on dental and oral health behaviors in female students aged 9–12 years old in the city of Rudsar, Guilan, Iran.

**Methods:**

This research is an interventional study conducted on 84 female students aged 9–12 years old, who lived in the city of Rudsar (*n* = 42 in the control group and *n* = 42 in the interventional group) in 2019. The data collection tools included questions on demographic variables, structures of the health belief model (perceived sensitivity, perceived severity, perceived barriers and benefits, self-efficiency), behavioral intention, and performance. The questionnaire was completed before the intervention and 3 months after it by both groups. The intervention group received four 45-min sessions. The data were analyzed using SPSS 24, descriptive tests, independent sample t-test, pair sample t-test, and regression (*P* < 0.05).

**Results:**

The mean age of the intervention and control groups was 10.88 ± 1.01 and 10.80 ± 1.01, respectively. The results showed that the average scores of all structures of the health belief model and behavioral intention in the intervention group significantly changed compared to the average scores obtained before the intervention (*P* < 0.05). Moreover, the average scores of perceived sensitivity (*p* < 0.009), perceived barriers (*p* < 0.007), self-efficiency (*p* < 0.001), and behavioral intention (*p* < 0.001) significantly changed after the intervention in both groups (*p* < 0.05).

**Conclusion:**

According to the results, the health belief model and the behavioral intention were effective in improving dental and oral health so that they can be applied to improving people's dental and oral health. It can also be used as a model to design, implement, and monitor medical health programs.

**Supplementary Information:**

The online version contains supplementary material available at 10.1186/s12903-022-02552-0.

## Background

Oral hygiene is an essential part of a person's general health, quality of life, and general well-being [1]. Dental and oral diseases are one of the most common diseases in the world and have serious health and economic burden, and severely reduce the affected people's quality of life [2]. The Global Burden of Disease (2017) showed that 3.5 billion people worldwide suffered from oral diseases and untreated tooth decay was the most common non-communicable disease [3]. Today, the prevalence and distribution of dental and oral diseases are different around the world [4]. According to the dental and oral health data of the World Bank, the prevalence of tooth decay in different countries ranges between 49 to 83% [5]. 60 to 90% of school students and almost 100% of adults have tooth decay. 61% of 6–12 years old children have one decayed and one fallen tooth in European countries [6]. The prevalence of tooth decay in Iran is high [7]. About 50% of 12-year-old children have tooth decay. And the mean of decayed, missing, and fallen teeth turned out to be 1.2, 0.06, and 0.23, respectively in iran [8]. The mean (SD) value of total DMFT index^1^ was 7.33 ± 3.0 in people aged 15–40 yr old of Kurdistan, western Iran in 2015 [9].

Until 2020, the new goals of the World Health Organization have focused on the use of experiences, evaluation of goals, and the importance of dental and oral health as an integrated part of general health [10].

Oral diseases are a major global public health problem. However, dental services have not yet health with such problems. On the other hand, dental services are not available and affordable for many people living in middle and low-income areas, especially in rural areas [11]. The consequences of untreated chronic oral and dental diseases are severe for the person and can include unrelenting pain, sepsis, reduced quality of life, loss of school days, disruption of family life, and reduced work productivity. Describing the prevalence and consequences of oral diseases and their neglect in global health politics, the urgent need to address oral diseases among other non-communicable diseases, as a global health priority [2]. Several public health initiatives have been implemented to control and reduce dental decay in children. The initiatives include providing free fluoride toothpaste, preschool brushing, oral hygiene programs, increasing access to affordable dental care, and school-based fluoride varnish programs [12]. It is also one of the main programs of the WHO in the field of chronic disease prevention and health promotion[13]. In this regard, there is an increasing focus on training dental hygiene and promotion programs to effectively control the progression of dental caries [12]. Training and dental and oral health self-care behaviors (flossing and brushing) can be mentioned as factors affecting reduced tooth decay [14–16]. The training interventions are useful for dental and oral health and are considered the first step in prevention and health culture promotion [17]. The first step in designing a training program is selecting a training model because "theories" in health education describe relationships and lie in the basics, through which the consequences and results of interventions can be predicted, measuring the effectiveness of the intervention [18]. Studies have shown that self-efficacy, perceived barriers, perceived sensitivity, and perceived severity are the most predictive factors of dental and oral health behaviors [19, 20]. These factors are the structures of the health belief model (HBM) in training health. This model is used to investigate the reasons for rejecting health issues by people and explain the behaviors of people who think they never get sick [21]. In the present study, the structure of behavioral intention taken from the theory of rational action and the theory of planned behavior will be examined as a structure before the structure of behavior in HBM. The second structure of rational action theory and planned behavior theory is behavioral intention. This structure includes thinking to perform a behavior, which is the immediate determinant of a specific behavior. It seems that there is a high consistency between intention and behavior. Individuals' intentions change over time. Therefore, behavior intention should be assessed sometime after the intervention [22]. Finally, according to studies and the researcher's inference, and the above-mentioned explanations, it seems that the health belief model is deficient in this regard. Given that the above-mentioned advantages refer to the lack of a structure in the health belief model, which would help measure the effectiveness of interventions if added to the model, the researcher suggests adding such a structure to the model. According to the Iranian Ministry of Health, most Iranian students do not pay enough attention to their oral health; 76.9% of them have reported that they brush less than twice a day. The highest age at risk of developing oral diseases is in the age range of 12–14 years old. Oral health problems can be still observed in this target group. This age range is of great importance because children finish their studies in elementary school and move to the higher level. Therefore, in many countries, this is the last station to reach a reliable sample through the school system [23]. Many studies have indicated that beliefs and attitudes about dental and oral health affect oral health-related behaviors [24, 25]. Considering the increasing incidence and prevalence of tooth decay, and the importance of the mentioned issues, as well as the characteristics and effectiveness of interventions based on the health belief model in changing behavior, the importance of self-care in dental and oral diseases and the importance of behavioral intention, the present study aimed to determine the effect of training based on health belief model and behavioral intention on self-care behavior in Iranian female students aged 9–12 years old. It is hoped to achieve improvement in students’ dental and oral self-care behaviors using the health belief model and adding the structure of behavioral intention from the rational action model and theory of planned behavior.

## Methods

The present study was a semi structured intervention conducted in 2019. The population consisted of students in the 3^rd^, 4^th^, and 5^th^ grades in elementary schools in the city of Rudsar. The samples were selected from schools of Rudsar using the cluster sampling method. This method was considered to be suitable because there was no list of all observations in many areas, as well as due to heterogeneity and classification in observations, and creating the distribution of economic, social, gender, educational level, etc. To select a sample of 84 students, 2 elementary schools were randomly selected, and then 2 classes were randomly selected from each school.

In this study, G*POWER was used to determine the sample size, and it was calculated to be 42 people in each group, considering the 5% first type error level, test power of 80%, and effect size of 60%.

In this study was used block randomization Permuted In which the size of the blocks was randomly selected so that 8 and 10 blocks (6 blocks of 10 and one block of 8) using Random allocation software were used and in each block there was an equal number of each group in which by blinding method Provided to the researcher.

**Fig. 1:**
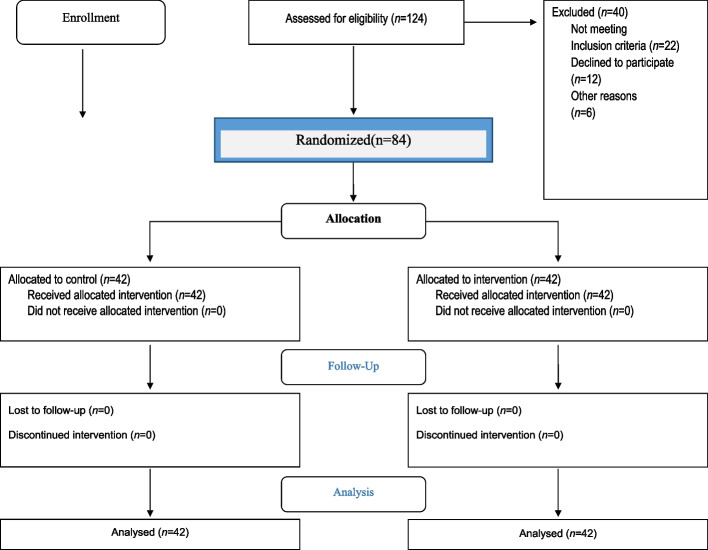
Flow chart of the study

The two groups were homogenous in terms of demographic variables. The inclusion criteria in the first stage included students in 3^rd^, 4^th^, and 5^th^ grades of elementary school, normal, aged 9–12 years, and willingness of students or their parents to participate in the study. The exclusion criteria were immigration, dropping out of school, and absence for more than one session. There were two groups in this study: a control group that received no intervention, and the intervention group that received the training program using the structures of the health belief model with the structure of behavioral intention. Figure 1 presents the study flow chart.

Before the intervention, the participants and their parents were explained about the purpose of the study, and the informed written consent forms were obtained. In addition, the training program for the intervention group is based on a model-based training package (speech, question and answer, discussion group, watching videos, pictures, and PowerPoint files) in four 45-min sessions. The subjects learned about the dental self-care program and its effect on dental and oral disease prevention, the importance of participation in training sessions, and the importance of the regular dental visit.

In a session, a 12-year-old student, who had tooth decay and loss due to a lack of dental and oral self-care, was asked to talk about her problems. A dentist was also asked to visit the students. In the end, a pamphlet was provided to the samples of the intervention group, and a WhatsApp group was considered for communication. The intervention group received the training program for 4 sessions. Every session took 45 min, and two follow-up sessions (one month and two months after the intervention) were held. Three months after the intervention, the data were gathered using the same questionnaire. Finally, the data were analyzed and compared.

### Data collection tools

The data collection tools in this study were a questionnaire, consisting of demographic characteristics, questions on perceived sensitivity, perceived severity, perceived benefits, perceived barriers, and perceived self-efficiency, taken from the health belief model, and the structure of behavioral intention derived from the rational action model and theory of planned behavior. It also included questions on the scale of performance concerning dental and oral health. The questions were scored using a 5-point Likert scale. The questionnaire and face-to-face interviews with the subjects were used to collect data.

To design the questionnaire, the questionnaire on the effect of the health belief model on students' dental and oral health developed by Dr. Parisa Kasmaei [26] was used and the questionnaire developed by Dr. Amir Pakpour Hajibaba [27] was used to assess the structure of behavioral intention. The validity and reliability of both questionnaires were confirmed [26, 27].

The questionnaire is composed of 2 parts:

- demographic characteristics including birth rank, family size, parents' education, parent's occupation, and living place.
- the structure of the health belief model and the structure of behavioral intention derived from the rational action model.
  1. The structure of perceived sensitivity included 3 items divided by a 5-point Likert scale. The lowest score had 0 points and the highest score had 5 points. The students' perceived sensitivity scores were scaled in three levels (low (1–5), medium (5–10), and high (10–15)).
  2. The structure of perceived severity included 7 items divided by a 5-point Likert scale. Zero point was deducted from the lowest score and 5 points to the highest score. The scores were scaled in three levels (low (0–11), medium (11–23), and high (23–35)).
  3. The structure of perceived benefits included 3 items divided by a 5-point Likert scale. The lowest score had 0 points and the highest score had 5 points. The scores were scaled in three levels (low (1–5), medium (5–10), and high (10–15)).
  4. The structure of perceived barriers included 7 items divided by a 5-point Likert scale. The lowest score had zero points and the highest score had 5 points. The scores were scaled in three levels (low (0–11), medium (11–23), and high (23–35)).
  5. The structure of self-efficiency included 5 items divided by a 5-point Likert scale. The lowest score had zero points and the highest score had 5 points. The scores of students' self-efficiency were scaled in three levels (low (0–8), medium (8–17), and high (17–25)).
  6. The structure of behavioral intention included three items divided by a 5-scale. The lowest score had zero points and the highest score had 5 points. The scores were scaled in three levels (low (1–5), medium (5–10), and high (10–15)).
  7. The questions on performance: two 7-part questions about brushing and Flossing times. The lowest score had zero points and the highest score had 7 points. The scores were scaled in three levels low (1–4), medium (4–9), and high (9–14)( Additional file 1: Appendix 1).

To analyze the data, a regression test was used to predict the variables. In this regard, the added structure of "behavioral intention" remained with the confirmation of the presence of the structure in the model. In general, the semi-structured study was performed when the results of the descriptive stage were determined.

In the second stage (semi-structured study), an independent sample t-test was used for double comparisons, and the repeated measure was used for repeated comparisons. The analyses were performed by SPSS.

## Results

The mean age of the intervention and control groups was 10.88±1.01 and 10.80±1.01. In terms of demographic variables, the target group had the highest birth rank (2), family size (2), father's education (diploma), mother's education (high school), mother's occupation (housewife), father's occupation (worker), and living place (private). There was no significant difference between the intervention and control groups in demographic variables. Therefore, the two groups were homogenous (Table 1).

**Table 1:** Homogeneity of demographic variables of the intervention and control groups (*n* = 84)

Demographic variables		Intervention group (*n* = 42) Frequency %	control group (*n* = 42) Frequency %	*P*value*
**birth rank**	1	18	15	*p* = 0.392
	2	23	21	
	3	1	2	
	4	0	2	
	5	1	3	
**Family size**	1	15	13	*p* = 0.370
	2	23	24	
	3	2	2	
	4	1	2	
	5 and above	1	2	
**Mother's education level**	illiterate	2	3	*p* = 0.169
	elementary	3	2	
	guidance	5	4	
	High school	3	5	
	diploma	17	14	
	Associate Degree	6	7	
	Bachelor's degree and higher	7	8	
**Father's education level**	illiterate	2	3	*p* = 0.343
	elementary	4	2	
	guidance	2	3	
	High school	12	11	
	diploma	13	15	
	Associate Degree	6	5	
	Bachelor's degree and higher	4	4	
**mother's job**	housewife	26	28	*p* = 0.740
	Employee	6	4	
	freelance(self-employed)	10	7	
	Other jobs	1	4	
**Father's job**	Unemployed	14	12	*p* =
	Employee	7	5	
	manual worker	15	17	
	free	7	9	
**place of residence**	Personal	35	29	*P* = 0.217
	rental	8	14	

* *p*<0/05

The results of the regression analysis indicated that the score of the HBM model structures could significantly explain the variance of the behavioral intention structures. The amount of explanation of the dimensions of the HBM model structures for the behavioral intention structure was found to be 56.3%. Therefore, the level of explanatory power of the scale of the HBM model structures is at a strong level. Also, the results showed that the structures of the HBM model have a positive and significant effect on the structure of behavioral intention. Based on the standard regression coefficient, the structure of perceived sensitivity benefits had the greatest effect (β=.340) and the structure of behavior had the least effect (=.205β) on the structure of behavioral intention (Table 2).

**Table 2:** Regression model of the influence of HBM model structures on behavioral intention structure

Factors	β coefficients		Std. Error	t	*p*-value	95% Confidence interval	
	Un standardized	standardized					
						Lower Bound	Upper Bound
Constant	56.98	-	.89	63.96	< .001	54.23	59.74
Perceived sensitivity	.390	.227	.040	9.818	< .001	.371	.405
Perceived intensity	.620	.302	.040	15.148	< .001	.498	.830
Perceived benefits	.362	.340	.041	8.775	< .001	.201	.513
Perceived barriers	.519	.213	.041	12.801	< .001	.387	.724
self-efficacy	.403	.226	.044	9.184	< .001	.235	.629
behavior	.589	.205	.044	13.441	< .001	.354	.781
Summary of the model	F = 81.794, *P *< .001		R-square = .572		adjusted- R-square = .563		

In the intervention group, the structures of the health belief model and behavioral intention showed significant differences from the results obtained before the intervention (3 months ago) while there was no significant difference in the control group. Also, there was found no significant difference between the two groups in the structures of the health belief model and behavioral intention before the intervention while, after the intervention, there was a significant difference between the two groups in the structures of perceived sensitivity, perceived barriers, self-efficiency, and behavioral intention (Table 3).

**Table 3:** Comparison of the structures of the health belief model and behavioral intention before and 3 months after the intervention in the two groups (*n* = 84)

structures of	intervention stage	control(*n* = 42)	intervention(*n* = 42)	*p*value*
**Perceived sensitivity**	Before intervention	7.72 ± 3.73	6.93 ± 2.61	*p* = 0.31
	After the intervention	7.85 ± 3.37	8.32 ± 3.15	*p* = 0.009**
	Pvalue**	*P* = 0.74	*p* = 0.001*	
**Perceived intensity**	Before intervention	11.51 ± 5.89	6.13 ± 14	*p* = 0.56
	After the intervention	12.34 ± 5.57	12.02 ± 4.39	*p* = 0.275
	*P*value**	*p* = 0.07	*p* = 0.004*	
**Perceived benefits**	Before intervention	4.13 ± 1.75	3.52 ± 0.80	*p* = 0.58
	After the intervention	24.27 ± 6.66	27.14 ± 10.87	*p* = 0.58
	*P*value**	*p* = 0.38	*p* = 0.05*	
**Perceived barriers**	Before intervention	25.56 ± 11.06	28.79 ± 4.39	*p* = 0.26
	After the intervention	3.97 ± 1.29	4.20 ± 1.82	*p* = 0.007**
	*P*value**	*p* = 0.68	*p* = 0.001*	
**self-efficacy**	Before intervention	7.97 ± 3.99	8.80 ± 3.74	*p* = 0.69
	After the intervention	8.58 ± 4.70	9.74 ± 4.97	*p* = 0.001**
	Pvalue**	*p* = 0.14	*p* = 0.003*	
**behavioral intention**	Before intervention	6.71 ± 2.70	7.02 ± 2.84	*p* = 0.74
	After the intervention	7.09 ± 2.75	7.46 ± 2.75	*p* = 0.001**
	Pvalue**	*p* = 0.46	*p* = 0.001*	
**behavior**	Before intervention	8.75 ± 3.34	9.11 ± 3.37	*p* = 0.38
	After the intervention	8.85 ± 2.60	9.70 ± 3.12	*p* = 0.69
	*P*value**	*p* = 0.12	*p* = 0.05*	

independent sample T-test(*p* < 0.0 5*)

^** ^Paire Sample T-test(*p* < 0.05*)

Table 4 shows the relationship between the demographic variables ad the structure of the health belief model and behavioral intention. There was a significant difference between the variables of birth rank, family size and Father's education with the structure of behavioral intention, and the variable of Father's education with the structures of perceived severity (Table 4).

**Table 4:** The relationship between demographic variables and the structure of the health belief model and behavioral intention

Model structure	Perceived sensitivity	Perceived barriers	Perceived benefits	Perceived intensity	Self efficacy	behavioral intention	Behavior
demographic variables							
Birth rank	0.071	0.751	0.500	0.810	0.284	0.004*	0.417
Family size	0.801	0.328	0.331	0.988	0.140	0.045*	0.098
Mother's education	0.185	0.337	0.232	0.466	0.160	0.328	0.518
Father's education level	0.502	0.052*	0.952	0.728	0.079	0.049*	0.724
Mother's job	0.927	0.248	0.139	0.317	0.503	0.840	0.822
Father's job	0.452	0.155	0.577	0.284	0.856	0.413	0.718
Place of residence	0.182	0.256	0.842	0.111	0.305	0.070	0.819

**regression (*p* < 0/0)**

## Discussion

The present study aimed to improve dental and oral health behaviors in Iranian female students aged 9-12 years old by performing a training program based on the health belief model and behavioral intention. The comparison of mean scores of the health belief model structures before and after the intervention showed a significant difference between the mean scores of the intervention and control groups, which was in line with the results of Sanaeinasab et al., (2022) [28], Jeihooni et al., (2022) [29], Noori Sistani et al., (2022) [30]. An HBM-based training program can be more effective than the programs currently used for training optimal health behaviors in children. Such interventions along with other school programs for tooth decay prevention can be useful for primary school children [28]. Shaghaghian suggested interventional methods, especially training programs for children and parents [31]. In this study, a unique, Specific and academic training program based on a model-based training package (speech, question and answer, discussion group, watching videos, pictures, and PowerPoint files) was performed for the intervention group in four 45-minute sessions. The subjects learned about the dental self-care program and its effect on dental and oral disease prevention, the importance of participation in training sessions, and the importance of the regular dental visit , which has caused the impact of training on improving the structures of the health belief model.

The comparison of mean scores of behavioral intention before and after the intervention showed a significant difference between the two groups, which was in line with the results of Zareban et al., (2021) [32], Shitu et al., (2021) [10], and Karimi et al., (2020) [33]. In other words, the results of the present study indicated that training and encouraging individuals to dental and oral self-care affect their behavioral intention for dental and oral self-care. Considering that in the present study, based on the results of the regression analysis, the added behavioral intention to the health belief model was consistent with all constructs. It seems that in the current study, significant changes in other structures of the health belief model [Perceived sensitivity, Perceived intensity, Perceived benefits, Perceived barriers, self-efficacy] in the field of promoting oral and dental health behavior have led to a significant increase in behavioral intention.

A person's intention to perform a certain behavior is a reflection of the person's motivationIt is to perform a behavior, in other words, does a person show the necessary desire to perform a certain behavior or not? [34]. One of the limitations of the study is the measurement of oral and dental health behavior using the participants' answers to the behavior questions of the research questionnaire, which not using the experimental evidence of oral and dental health [such as dental plaque, the amount of decay or filling tooths of the participants before and after intervention]. The relationship between behavioral intention and behavior showed that people tend to be involved in behaviors they have intended to do; therefore, behavior is always the result of intention and associated with it [35]. In the other hand the behavior is controlled by behavioral intention and other possible factors might indirectly influence behavior through behavioral intention. behavioral intention is an individual’s intention to engage in certain behavior [36]. The behavioral intentions, the immediate precursors of behavior, are determined by attitude toward the behavior and used widely for the prediction and modification of human actions [37]. The structure of behavioral intention plays an important role in the formation of preventive behavior, and if the mentioned factor is subjected to appropriate training and intervention in educational interventions, a higher explanation of the intended behavior can be expected [34]. So Adding behavioral intention to the health belief model can be used to measure the impact of the educational intervention on the oral health behavior of the participants and can be considered as a criterion for their verification, and on the other hand, it can be a suitable step in the direction of adjusting the HBM model.

The results of present research show that, the values of perceived susceptibility and perceived severity (perceived threat) of experimental group has significant enhancement. Indicating the effectiveness of intervention in improving perceived susceptibility and perceived severity of the experimental group, The educational intervention for experimental group is performed in educational sessions by giving model-based training package (speech, question and answer, discussion group, watching videos, pictures, and PowerPoint files) that increased the perceived sensitivity in people. So In a session, a 12-year-old student, who had tooth decay and loss due to a lack of dental and oral self-care, was asked to talk about her problems that increased the perceived severity in people.That is consistent with the results of Ashoori et al. (2021) [38], Ghaffari et al. (2018) [39] and Ismael et al. (2019) [40].That indicates the effectiveness of the performed intervention program in increasing perceived sensitivity and perceived severity, as the factors affecting the behavior with a deficiency that should be taken into consideration by school teachers and health educators. Adolescents may realize the critical point of health situations if they do not feel vulnerable to them, so educators should help them deal with reality.

The results of this study show significant enhancement in average score of perceived barriers in experimental group. That is consistent with the results of Vaezipour et al. (2018) [41], Phanthavong et al. (2019) [42] And Moore et al. (2022) [43]. Among barriers to oral health are barriers related to the mother and family members (attitudes, inappropriate behaviors, and skills, mental, emotional, and knowledge conditions) as well as the barriers related to the child (skill), lack of dental health services, and dental knowledge [44–46]. In a study by Oveisi et al., [2019], perceived barriers were one of the strongest and most important predicting factors of students' behavior, which should be considered in planning school curricula [47]. According to the results of the present study, perceived barriers significantly decreased after the intervention in the intervention group compared to the control group. After the educational intervention, experimental group learn about the benefits of preventive behaviors from dental health and have less barriers for taking these behaviors. In current study, presenting educations about dental health and educational pamphlet help the increase of perceived benefits and dominating on barriers.Therefore, the model can be used as an effective model in decreasing perceived barriers to school students' oral health.

The results of this study show significant enhancement in average score of Perceived benefits in experimental group. That is consistent with the results of Noori Sistani et al. (2022) [30], Xiang et al. (2022) [48] AND Rahimzadeh et al. (2022) [49]. According to the results of Taheri et al., (2021), one of the most important predicting factors of oral health in students aged 10–12 years old was perceived benefits in the HBM model [50]. Perceived benefits are defined as the person's belief in the efficiency of the measures suggested to decrease the risk or threat [49]. It seems that, in the present study, the subjects learned about the dental self-care program and its effect on dental and oral disease prevention, the importance of participation in training sessions, and the importance of the regular dental visit that significantly increased the score of perceived benefit structure in the intervention group after they were explained the role of dental self-care behaviors in decreasing dental problems and tooth decay.

The results of this study show significant enhancement in average score of Self-efficacy in experimental group. which shows the effect of the educational intervention on the self-efficacy of the students, that is, the education of the students of the intervention group made them believe that they can take good care of their teeth and use the health recommendations efficiently. That is consistent with the results of Goodarzi et al. (2019) [23], Oveisi et al. (2019) [47] and Asawa et al. (2020) [51]. Self-efficiency refers to an individual's belief in his or her capacity to execute behaviors [49]. Students' belief in their capacity to execute appropriate dental and oral health behaviors can be effective in improving their self-efficiency. The structure of self-efficiency can be strongly associated with the incidence of a behavior; indeed, it is the cause of a behavior, which should be taken into consideration. An HBM-based interventional program can help improve self-efficacy and adopt useful dental self-care behaviors.

The results of this study show significant enhancement in average score of Self-care practice(behavior) in experimental group. That indicating the effect of education on student s’ dental health behavior after the educational intervention. That is consistent with the results of Asawa et al. (2020) [51], Kazemi et al. (2020) [52] and Aurlene et al. (2020) [53] studies. Therefore, it is suggested to use appropriate training interventions along with behavioral models and theories, such as the health belief model, to increase self-care performance and improve dental and oral health in students.

There was a significant relationship between the variable of family size and Father's education with the structure of behavioral intention, and the variable of Father's education with the structures of perceived severity, which was in line with the results of Dumitrescu(2014) [54]، Xiang (2022) [55] studies. According to what was discussed, a health behavior in the health belief model depends on two issues: first, the person's perception of the risk threatening him/her, and second, the perceived barriers and benefits of behavior. On the other hand, the relationship between behavioral intention and behavior showed that people tend to be involved in behaviors they have intended to do. In addition to the mentioned factors, appropriate health behavior is influenced by other factors such demographic characteristics of the student and her parents. The results showed that while designing school-based dental self-care interventional programs, the demographic characteristics of students and their parents, attitudes (perceived sensitivity, perceived severity), and students' behavioral intentions should be specifically considered. On the other hand, training elementary students is not enough, training parents and their participation along with new considerations to improve students' dental and oral health behaviors is an integrated part of the program. Self-report in health behaviors and lack of generalization of the results to other age and demographic groups can be mentioned as limitations of the present study. Therefore, it is suggested for further studies research other groups of participants. enother limitations, including the fact that Demographic characteristics of students and their parents are the factors affecting the dental health of students that was not under the control of the researcher.. Also,researchers don’t had the follow-up of the clinical effect[such as dental plaque, the amount of decay or filling tooths of the participants before and after intervention] of the current intervention in the two intervention groups and the control group at two time intervals before and 3 month after the intervention is another limitations of the present study. This debate has been somewhat alleviated by adding behavioral intention to the health belief model.

The strengths of the study were studying the vulnerable groups of children, being problem-based, and using the integrated behavioral model (HBM and the structure of behavioral intention in TPB) to improve students' dental self-care.

## Conclusion

The results indicated that health training based on the health belief model and behavioral intention had a significant effect on increasing the scores of the health belief model and behavioral intention. Therefore, the Education authorities are required to adopt theory-based training programs to create and improve dental and oral self-care behaviors in students. Creating a culture of dental and oral health is one of the necessities in society. As the rates of tooth decay indicate in Iran and the world, the dental and oral health culture needs knowledge and awareness, which should be considered more in elementary schools. Presenting knowledge and information about the importance of oral health at a wider level of society, the media can be an important source of knowledge to communicate health messages to the public and play an effective role in improving dental and oral health. Students will live happier, more lively, and more physically and mentally healthy if they have healthy teeth. Thus, they should learn how to take care of their teeth. In this regard, theory-based educational materials on dental and oral self-care in the school curriculum can be beneficial.

## Supplementary Information

**Additional file 1:** **Appendix 1. **Questionnaire.

## Data Availability

The datasets used and/or analysed during the current study available from the corresponding author on reasonable request.

## Abbreviations

DMFT: Decayed Missing and Filled Teeth
HBM: Health Belief Model
TPB: Theory of planned behavior

## Footnotes

1 .DMFT index = Decayed (D), Missing (M), and Filled teeth (FT).
