# Supplementary material for: Effect of training based on health belief model and behavioral intention on improving dental and oral self-care behavior in 9–12-year-old Iranian female students
Source: BMC Oral Health. 2022 Nov 19;22:515. doi: 10.1186/s12903-022-02552-0 (PMC9675157; doi:10.1186/s12903-022-02552-0)
Supplement: Supplementary file 1 — Additional file 1: Appendix 1. Questionnaire. [file 12903_2022_2552_MOESM1_ESM.docx]

**Appendix 1- Questionnaire**

**IN THE NAME OF GOD**

**Hello, my dear friend, thank you very much for helping me fill out this questionnaire.**

**1. Name of the school: …..**

**2.Questionnaire number:**  …..

**A-Demographic questions:**

**3. birth rank: …..**

**4.Family size: …...**

**5.** **Do you have any high school brothers or sisters? (If the answer is yes, their gender):….**

**6.Mother's education level:** illiterate🞏 elementary🞏 guidance🞏 High school🞏diploma🞏 Associate Degree🞏 Bachelor's degree and higher🞏

**7.Father's education level:** illiterate elementary guidance High schooldiploma Associate Degree Bachelor's degree and higher

**8.mother's job:** housewife Employee freelance(self-employed) Other jobs

**9.Father's** **job:** Unemployed **** Employee freelance(self-employed) Other jobs

**10. place of residence:** Personal**** rental

**B- structure of behavioral intention and the structures of the health belief model questions:**

| **Perceived sensitivity:** | **completely agree** | **agree** | **neither agree nor disagree,** | **disagree** | **completely disagree** |
| --- | --- | --- | --- | --- | --- |
| 11- As long as I am young, my teeth remain healthy. |  |  |  |  |  |
| 12- My teeth are healthy because none of them hurt. |  |  |  |  |  |
| 13- My teeth are strong, so they stay healthy. |  |  |  |  |  |

| **Perceived intensity:** | **completely agree** | **agree** | **neither agree nor disagree,** | **disagree** | **completely disagree** |
| --- | --- | --- | --- | --- | --- |
| 14- The decay of my teeth makes me have a toothache. |  |  |  |  |  |
| 15- If my teeth decay, I may lose them. |  |  |  |  |  |
| 16- The decay of my teeth makes my mouth smell bad. |  |  |  |  |  |
| 17- The decay of my teeth causes me to spend a lot of money for treatment. |  |  |  |  |  |
| 18- The decay of my teeth makes me look ugly when I laugh. |  |  |  |  |  |
| 19- I cannot eat well with decayed teeth. |  |  |  |  |  |
| 20- If I lose my teeth, I won't be able to speak well. |  |  |  |  |  |

| **Perceived benefits** | **completely agree** | **agree** | **neither agree nor disagree,** | **disagree** | **completely disagree** |
| --- | --- | --- | --- | --- | --- |
| 21- Brushing can prevent my teeth from decaying. |  |  |  |  |  |
| 22- Brushing prevents bad breath. |  |  |  |  |  |
| 23- Maintaining oral and dental hygiene makes me able to eat better. |  |  |  |  |  |

| **Perceived barriers** | **completely agree** | **agree** | **neither agree nor disagree,** | **disagree** | **completely disagree** |
| --- | --- | --- | --- | --- | --- |
| 24- My gums hurt when I brush my teeth. |  |  |  |  |  |
| 25- My gums bleed when I brush my teeth. |  |  |  |  |  |
| 26- Brushing my teeth takes my time. |  |  |  |  |  |
| 27- I don't brush my teeth if I'm tired. |  |  |  |  |  |
| 28- I am lazy in brushing my teeth. |  |  |  |  |  |
| 29- Sometimes I don't feel like brushing my teeth. |  |  |  |  |  |
| 30- Sometimes I forget to brush my teeth. |  |  |  |  |  |

| **self-efficacy** | **completely agree** | **agree** | **neither agree nor disagree,** | **disagree** | **completely disagree** |
| --- | --- | --- | --- | --- | --- |
| 31- I can brush my teeth even if I am tired. |  |  |  |  |  |
| 32- I can brush my teeth properly. |  |  |  |  |  |
| 33- I can continue brushing for the necessary time (at least 2 minutes) each time. |  |  |  |  |  |
| 34- I can brush my teeth even if I have an exam. |  |  |  |  |  |
| 35- I can brush my teeth regularly even if my parents don't remind me. |  |  |  |  |  |

| **behavioral intention** | **completely agree** | **agree** | **neither agree nor disagree,** | **disagree** | **completely disagree** |
| --- | --- | --- | --- | --- | --- |
| 36- I plan to set a specific time for brushing. |  |  |  |  |  |
| 37. I plan to floss all my teeth. |  |  |  |  |  |
| 38-I want others to know about my brushing. |  |  |  |  |  |

**Behavior:**

**39- How often do you brush your teeth?**

1- Never 2- Once every two or more weeks 3- Once a week 4- Twice a week 5- Every other day 6- Once a day 7- Twice a day or more

**40- How often do you floss all your teeth at once?**

1- Never 2- Once every two or more weeks 3- Once a week 4- Twice a week 5- Every other day 6- Once a day 7- Twice a day or more

**My dear friend, do not be tired, thank you very much for your kindness.**

**Be happy, healthy and successful.**
